# Alpha-to-beta cell trans-differentiation for treatment of diabetes

**DOI:** 10.1042/BST20210244

**Published:** 2021-12-09

**Authors:** Mohamed Saleh, George K. Gittes, Krishna Prasadan

**Affiliations:** 1 Division of Pediatric Surgery, UPMC Children's Hospital of Pittsburgh, Pittsburgh, PA 15224, U.S.A.; 2 Division of Pediatric Endocrinology, UPMC Children's Hospital of Pittsburgh, Pittsburgh, PA 15224, U.S.A.

**Keywords:** alpha cell, diabetes, gene therapy

## Abstract

Diabetes mellitus is a significant cause of morbidity and mortality in the United States and worldwide. According to the CDC, in 2017, ∼34.2 million of the American population had diabetes. Also, in 2017, diabetes was the seventh leading cause of death and has become the number one biomedical financial burden in the United States. Insulin replacement therapy and medications that increase insulin secretion and improve insulin sensitivity are the main therapies used to treat diabetes. Unfortunately, there is currently no radical cure for the different types of diabetes. Loss of β cell mass is the end result that leads to both type 1 and type 2 diabetes. In the past decade, there has been an increased effort to develop therapeutic strategies to replace the lost β cell mass and restore insulin secretion. α cells have recently become an attractive target for replacing the lost β cell mass, which could eventually be a potential strategy to cure diabetes. This review highlights the advantages of using α cells as a source for generating new β cells, the various investigative approaches to convert α cells into insulin-producing cells, and the future prospects and problems of this promising diabetes therapeutic strategy.

## Introduction

Type 1 diabetes is a chronic disease characterized by autoimmune-mediated destruction of the insulin-producing β cells in the pancreas [1]. Destruction of β cells leads to insulin deficiency, hyperglycemia, and eventually the development of clinical diabetes. The treatment options for type 1 diabetes are limited to insulin replacement therapy. While, type 2 diabetes results from long-standing insulin resistance [2]. In type 2 diabetes, β cell mass is reduced by 40–60% compared with weight-matched controls [3,4]. The underlying etiology of β cell mass loss in type 2 diabetes is thought to be due to an increase in β-cell apoptosis rate [5], as chronic exposure to insulin resistance imposes a high workload on β cells in order to meet the higher demand for insulin secretion, which makes β cells vulnerable to endoplasmic reticulum (ER) stress [6,7]. Besides the decreased β cells in patients with type 2 diabetes, β-cell dysfunction also occurs early in the natural course of type 2 diabetes, with a decline in β cell function of 75–80% in subjects in the upper third of impaired glucose tolerance (2 h plasma glucose = 180–199 mg/dl) [6,8,9]. Additionally, reduced first-phase insulin secretion was found to be the earliest and most detrimental defect in β cells in humans with impaired glucose tolerance (prediabetes) and type 2 diabetes [10,11].

There is no cure for type 1 and type 2 diabetes. The prevailing therapeutic approaches to type 2 diabetes have focused on drugs that either improve insulin resistance or increase insulin secretion and decrease glucagon secretion [12]. However, these medications increase the risk of side effects such as dysregulated insulin secretion, weight gain, hypoglycemia, and gastrointestinal, renal, and cardiovascular side effects [13,14]. Although the pathophysiology of type 1 and type 2 diabetes are different, loss of β cell mass with subsequent insulin deficiency represents the end result that directly causes diabetes. Therefore, a better therapeutic strategy would be to enhance β cell mass and restore insulin secretory capacity, which might cure different types of diabetes.

## Key transcription factors involved in the development of β cells

**Figure 1.**
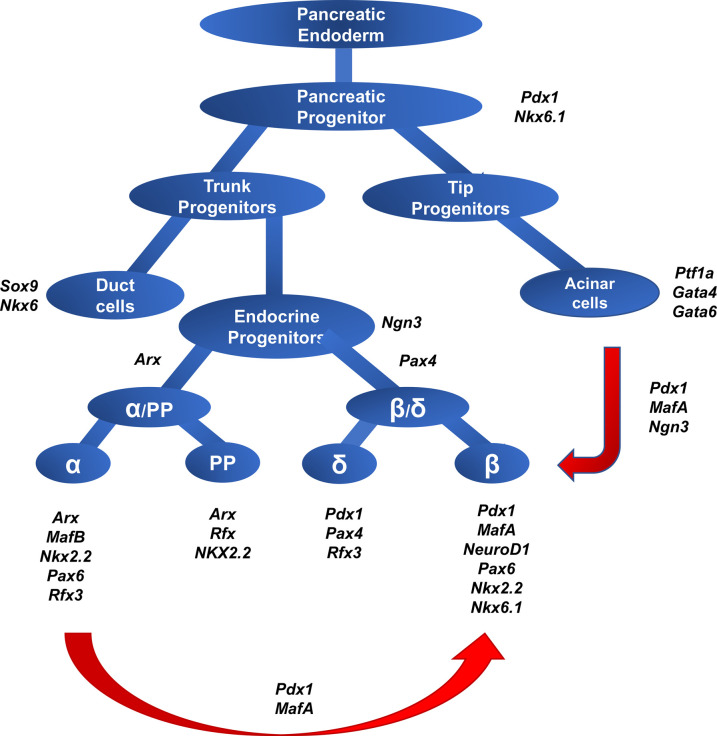
Mouse pancreas development originates from the foregut endoderm under the control of several transcription and growth factors, specifically *pdx1*. Loss of *pdx1* prevents the formation of the pancreas, while overexpression of endocrine-specific transcription factor, *ngn3,* in foregut endoderm will result in an immature pancreas containing only α cells. Loss of *ngn3* expression in these cells prevents endocrine development. *pdx1* positive progenitors develop both trunk and tip progenitors, *ngn3* expression in trunk progenitor then leads those into an endocrine lineage and generates all four endocrine cell types. *Pdx1* and *MafA* expression in select endocrine progenitors gave way to β cells, while *MafB* expression is required for α cell formation. Forced expression on *pdx1, MafA, and ngn3* in acinar cells reprograms them into β cells, while forced expression of *pdx1* and *MafA* in α cells converts them to β cells.

In the developing pancreas, cellular differentiation and lineage selection are regulated by a cascade of transcription factors and signaling molecules that coordinate the timing and development of the exocrine and endocrine cells from progenitor cells (Figure 1)*.* The differentiation of endocrine cell types in the pancreas changes throughout embryogenesis. Specifically, α cells are the first to form, with glucagon-positive cells appearing early in the developing mouse pancreas at E9.5, with a subset of these cells co-express insulin [15,16]. This finding suggests that pancreatic endocrine progenitor cells co-express a set of islet hormones whose expression is selectively up or down-regulated as the endocrine lineage selection occurs. Beyond α cells, other types of endocrine cells, including β cells, are not generated in significant numbers in mouse until E13.5 or later [17].

In mice, early embryonic *pdx1* positive cells represent progenitors of all of the mature endocrine and exocrine pancreatic cells [18]. *Pdx1* expression becomes limited to β cells late in the development of the murine pancreas as β cells mature [19]. Also, *pdx1* is known to regulate the insulin genes in rodents [20]. Besides, *PDX1* is required for normal β cell function, and loss of its expression from one allele in adult humans causes diabetes [21,22]. Conditional deletion of *pdx1* in the developing β cells in rodents results in hyperglycemia, reduced number of β cells and an increased number of α cells [23,24]. In the developing mouse pancreas, all endocrine cells develop from neurogenin-3 (*ngn3*) positive endocrine progenitor cells [18,25]. *Ngn3* [a class A Basic Helix-Loop-Helix Protein (bHLH)] is expressed in duct-like epithelial cells that are centrally located within the developing mouse pancreas; as these cells differentiate, they down-regulate *ngn3* and aggregate into proto-islet structures [26]. Loss of function mutation of *ngn3* prevents endocrine development and leads to death in mice postnatally [25,27–29]. Although it is critical for all pancreatic endocrine cell identity, forced expression of *ngn3* early during the mouse pancreas development under the *pdx1* promoter led to the formation of a premature cluster of endocrine cells containing only α cells [26], suggesting a role for other factors besides *ngn3* later in development for proper endocrine development and specification.

The Maf family proteins, *MafA*, and *MafB* have a central role in the late development and maturation of endocrine cells in rodents [30–32]. In the embryonic mouse pancreas, a significant portion of insulin-positive cells express *MafB*, and as part of the β cell maturation process, these cells transitioned through a *MafB* and *MafA* double-positive phase (insulin intermediate cells) followed by full maturation to a *MafB* negative and *MafA* positive β cells. This transition of β cells to become *MafA* positive only coincides with increased *pdx1* expression in these mature cells [33]. *MafB* (−/−) null mutant embryonic pancreas had reduced numbers of insulin and glucagon-positive cells, yet, the total number of endocrine cells appeared to remain the same [34,35]. Unlike mice, adult human islet β cells express *MAFB* [36]. Human progenitor stem cells lacking *MAFB* expression failed to differentiate into α or β cells, but formed delta and pancreatic polypeptide cells [36].

The *MafA* null mutant mouse showed that *MafA* is necessary for maturation but not for the specification of pancreatic β cells. [33]. Losing *MafA* during the development of mouse pancreas did not alter the proportion of insulin-positive cells at birth, suggesting normal development and lineage selection [33]. However, the MafA deficient mice developed diabetes postnatally, suggesting that *MafA* regulates maturation and is required for glucose-responsive expression of insulin in adult β cells [33]. To this point, *MafA* interacts with *NeuroD1* and *Pdx1* [29] to activate the insulin gene in mice [30]. Besides *Pdx-1* and *MafA*, in rodents, the mature β-cell expresses *Pax4*, *Nkx* 2.2, and *Nkx* 6.1, which are also required to maintain normal function [37].

## Non-endocrine cells as a source of new β cells

There have been several attempts in the past to convert non-endocrine cells into insulin-producing cells [38,39], including viral-mediated expression of transcription factors in human hepatocytes [38], mouse gastrointestinal cells [40], and acinar cells [41,42]. Ectopic *PDX1* expression in adult human liver cells induced the development of functional insulin-producing cells; these cells when transplanted under the renal capsule of diabetic, immunodeficient mice ameliorated hyperglycemia [43]. Also, *In vivo* recombinant-adenovirus-mediated gene transfer of *pdx1* into liver cells ameliorated hyperglycemia in diabetic mice treated with streptozotocin [44]. Furthermore, treatment with exendin-4 (Glucagon-like peptide agonist) enhanced the proliferation and maturation of *PDX1*-expressing human liver cells toward a β cell phenotype [45]. In addition, plasmid-based *pdx1, MafA*, and *ngn3* (PMN) gene delivery into the inferior vena cava transiently induced insulin transcripts in rat livers [46]. Also, systemic administration of a single adenoviral vector encoding *pdx1*, *MafA*, and *ngn3* factors reprogrammed duct-like SOX9-positive cells in the liver into insulin-producing cells and improved hyperglycemia in non-obese diabetic/severe combined immunodeficient (NOD/SCID) mice treated with streptozotocin [47]. Notably, those insulin-producing duct-like cells showed some degree of glucose responsiveness *ex vivo* [47]. In immunocompetent mice, adenoviral vector-mediated PMN delivery transiently induced insulin-producing SOX9-positive duct-like cells in the liver [48].

Acinar cells were another cell type targeted as a potential source of new insulin-producing cells. Forced expression of *ngn3* alone in mouse acinar cells induced conversion into delta cells, while forced *ngn3* and *MafA* expression converted acinar cells into α-like cells [41]. However, a combination of the three transcription factors, *ngn3*, *MafA* and *pdx1*, converted the acinar cells into beta-like cells and improved hyperglycemia in toxin-induced-diabetic mice [41,42].

Besides pancreas acinar cells and liver cells, several studies have targeted gastrointestinal cells as a potential source to form insulin-producing cells. The gastrointestinal tissue is abundant with adult stem/progenitor cells that are continuously forming epithelial cells, including enteroendocrine cells [49,50]. In fetal and adult mice, specific deletion of *FoxO1* in *ngn3*-positive enteroendocrine progenitors converted them into insulin-positive cells [51]. This ablation of the *FoxO1* in the enteroendocrine cells increased the expression of β-cell transcription factors *pdx1*, *ngn3*, *MafA* and *Nkx6.1* [51]. In human pluripotent stem (iPS) cells, *FOXO1* inhibition induced the generation of insulin-producing cells that express all markers of mature pancreatic β cells [52]. Similar to acinar cells and liver cells, forced expression of *ngn3*, *MafA* and *pdx1* in mice intestinal crypt cells and human intestinal organoids converted them to β-like cells [53]. In this study, expression of *ngn3*, *MafA* and *pdx1* in intestinal cells lead to modest but significant improvement in glucose tolerance in Streptozotocin-treated mice [53]. Similarly, forced expression of *ngn3*, *MafA* and *pdx1* reprogrammed gastrointestinal enteroendocrine cells to insulin-producing cells with the highest efficiency being observed in the stomach antrum [54].

One major problem with targeting non-endocrine cells as a source of new insulin-producing cells is that these attempts only resulted in a partial improvement in glycemia, specifically fasting glucose, in diabetic mouse models. The overall glucose tolerance, however, despite the improvement in fasting glucose levels, remained quite abnormal, indicating that the newly formed β-like cells can secret some basal insulin, but they cannot respond adequately to a glucose challenge, which obviously raises concerns about the potential translatability of this approach.

A second drawback with reprogramming a non-endocrine cell such as an acinar cell to become an insulin-producing cell (or any islet endocrine cell for that matter), *ngn3* is necessary to initiate an endocrine lineage identity and to suppress the acinar cell phenotype [41]. Subsequently, *pdx1* and *MafA* further convert *ngn3* positive cells into insulin-producing cells. Here, the continued expression of *ngn3* in differentiated islet cells is a significant drawback of this approach when trying to generate β cells from non-endocrine cells because *ngn3* expression is normally low or absent from differentiated islet endocrine cells [55]. Thus, the use of a triple transcription factor vector encoding *pdx1*, *MafA* and *ngn3* would lead to constitutive expression of relatively high levels of *ngn3* in the trans-differentiated β-like cells, which may have negative consequences. Thus, targeting endocrine cells seems a preferable approach to regenerating β/β-like cells.

## Why α cells may be an optimal source for new β cell formation

Recently, researchers have shifted their focus toward α cells as a source for the replacement of β cells. Several reasons favor α cells as a proper source for β cell replacement compared with non-endocrine cells, including: (1) α and β cell lineages appear to arise from a common precursor [15,56], which may facilitate reprogramming. (2) evidence already exists for the potential interconversion between α and β cells; postnatal deletion of *pdx1* in mouse β cells led to loss of β cells with an increase in α cells, accompanied by a change in islet morphology, with glucagon-positive cells in the periphery and center of the islet, rather than the usual periphery only in mice. In addition, some cells were double-positive for both glucagon and insulin [24]. Also, it is reported that a massive loss of β cells in the adult mouse pancreas led to the conversion of α cells into β cells, again with the appearance of bi-hormonal cells expressing both insulin and glucagon [57], additionally, monoclonal antibodies to glucagon receptor were found to induce α cell hyperplasia and subsequently α cells were converted to β-like cells [58], thus supporting the ability to reprogram α cells into insulin-producing cells therapeutically since it can happen spontaneously. (3) the similarities in the function of α cells and β cells, as both cells have Slc2a2 transporter that allows glucose sensing within a physiologic range [59]. Also, α cells and β cells have similar machinery to metabolize glucose and secrete hormones [60]. (4) α cells are located anatomically in the islet, receiving the same blood supply [15], additionally, in humans, α and β cells being located in islets, they receive sympathetic nerve supply through the splanchnic nerve with the neural cell bodies originate from the superior mesenteric and celiac ganglia, while the parasympathetic innervation comes from the vagus nerve [61], which is ideal for the optimal function of newly formed β-like cells [62]. (5) α cells represent ∼35% of the islet cells in humans [63], which makes them an abundant source for β cell replacement. (6) Reduction in α cell mass in mice does not have a negative impact on glucose metabolism [64]. In view of these reasons, α cell appears to be an ideal therapeutic target for replacement of β cells to treat diabetes.

## Therapeutic attempts to reprogram *α* cells into insulin-producing cells

Several attempts were made to reprogram α cells into insulin-producing cells *ex vivo* and *in vivo*.

For example, *in vivo* forced expression of *pax4* in mouse islet progenitors induced production of α-like cells that then converted into β-like cells, forming large islets with β cell predominance; *ngn3* reactivation was crucial in this process [65]. In this model, *the pax4* forced expression in islets not only increased β cell mass, but led to improved glucose tolerance. Furthermore, the ectopic expression of *pax4* in adult α cells continuously converted them into β cells and reversed hyperglycemia in streptozotocin-treated animals; interestingly, this effect was only seen in mice younger than four weeks [65].

Sangan et al. [66] reprogrammed an α cell line, αTC1.9, into insulin-producing cells by ectopic expression of HNF4α, which resulted in glucagon suppression and induced a β-like cell phenotype; however, that reprogramming was incomplete because certain β cell-specific transcription factors such as *pdx1* were not induced.

Similarly, Zhang et al. delivered *pax4* via a viral vector (adenovirus 5) into αTC1.9 cells leading to an induction of insulin synthesis and suppression of glucagon. Here, *pax4* expression led to an up-regulation of the β cell transcription factors *pdx1*, *MafA*, *ngn3,* and *nkx 6.1* in the αTC1.9 cells. Also, direct infusion of adenovirus 5 carrying a *pax4* expression cassette into the pancreas via the pancreatic duct resulted in a small improvement in glucose tolerance in toxin-induced diabetic mice, though not a biologically significant improvement [67].

Furthermore, inactivation of *arx* and *dnmt1* in adult mouse pancreatic α cells led to conversion of a subset (50–80% over three months) of these α cells into β-like cells with the capacity to secrete insulin in response to glucose stimulation, yet this insulin secretion capacity was significantly lower than true β cells [68]. Based on the results of that study, a follow up study used the anti-malaria drug artemether to suppress the α cell transcription factor *arx* in mice to promote trans-differentiation into β-like cells [69]. However, the key initial experiments in this study were carried out in islet cell lines, but subsequent validation experiments *in vivo* showed some degree of trans-differentiation, but without a clear demonstration of α to β cell conversion; moreover, artemether was found to abrogate β cell calcium signaling and insulin secretion in response to glucose [70].

In the same context, another study reported that the prolonged exposure of wild-type mice to GABA resulted in the conversion of α cells into β-like cells through the down-regulation of Arx expression [71]. In this study GABA treatment successfully reversed hyperglycemia in Streptozotocin-treated mice [71]. Also, Young-sun et al. have shown that glucagon-like peptide 1 promoted the formation of new β-like cells from α cells in mice via FGF21 after chemical ablation of β cells with Streptozotocin [72].

**Figure 2.**
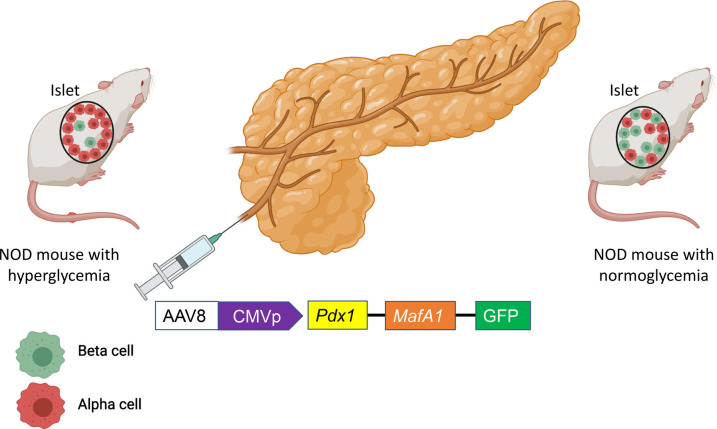
In NOD mice with hyperglycemia, pancreatic intraductal infusion of adeno-associated virus-containing *pdx1* and *MafA* converted α cells into β cells and restored normoglycemia. Thorel et al. [57] found similar α to β cell trans-differentiation de novo after an extreme loss of β cells. However, the conversion and the rescue process took very long time compared with the viral-mediated α to β cell trans-differentiation.

More recently, the focus has shifted to overexpression of *MafA* and *pdx1* in α cells to convert them into β-like cells. *In adult mice,* induced expression of *MafA* and *pdx1* in *ngn3* positive endocrine progenitor cells led to the development of a β-like cell phenotype; similarly, *pdx1* and *MafA* overexpression in α cells led to its trans-differentiation into β-like cells [73]. This latter study was followed by a study that used an *in vivo* infusion of adeno-associated virus (AAV) carrying *pdx1* and *MafA* expression cassettes into the pancreatic duct, leading to reprogramming of α cells into functional β-like cells with normalization of blood glucose in both β cell-toxin-induced diabetic mice and in autoimmune NOD mice (Figure 2). In that study, the euglycemia persisted in the autoimmune NOD mice for four months before the recurrence of hyperglycemia, perhaps because the immune system began to recognize and destroy the newly formed β-like cells. This gene therapy strategy also induced α to β cell conversion in toxin-treated human islets, which restored blood glucose levels in NOD/SCID mice upon transplantation [74].

A subsequent study sought to better study human islet cell plasticity, specifically the ability of human α cells to transform into β cells. *In vitro* infection of human α-cell-only pseudoislets with adenovirus expressing *PDX1* and *MAFA* led to conversion of ∼35% of these α cells into insulin-positive cells [75]. Moreover, transplantation of pseudoislets, made of α cells infected with this same *pdx1* and *MafA* adenovirus, into diabetic immunodeficient NOD/SCID/Il2rg^−/−^ (NSG) mice led to improved insulin secretion and glucose tolerance. The improvement fell short of full normalization, likely due to an inadequate mass of transplanted reprogrammed α cells [75].

## Α cell to β cell conversion; challenges and future directions

Translation of this therapeutic gene strategy to treat diabetes in humans seems technically applicable via the noninvasive procedure endoscopic retrograde cholangiopancreatography (ERCP). However, finding a proper viral vector that could carry the genes and targets human α cells with high affinity *in vivo* remains a challenge. Xiao et al. and Furuyama et al. used AAV serotype 8 virus to deliver *pdx1* and *MafA* to human α cells *ex vivo* [74,75]. However, directly infecting islets *ex vivo* with the virus differs significantly from using an *in vivo* pancreatic duct infusion. In cell culture, the virus is placed in direct contact with the islets, making the pathway to viral infection of the islets very different. *In vivo*, the virus must pass out of the pancreatic ductal system, crossing the pancreatic duct epithelium and ductal basement membrane, or crossing the acinar cells and the acinar basement membrane, across the interstitial space before finally reaching the islets, which also *in vivo* are surrounded by a basement membrane ‘capsule’ [76]. This capsule is degraded during the islet isolation process and therefore not a barrier to *in vitro* islet infection by virus. During this journey, undesirable trapping of the virus in exocrine cells may occur before they can reach the islets and potentially preventing adequate numbers of virus from reaching the α cells. Finding the ideal vector (virus type and serotype) for infecting α cells in humans will likely require further studies in non-human primates. This primate optimization will be necessary before persuing clinical trials in humans to ensure the safety of the viral therapy, minimizing the risk of adverse extrapancreatic side effects, and optimizing efficacy. Efficacy will entail infecting and reprogramming an adequate number of α cells into insulin-producing cells enough to reverse hyperglycemia.

The immunogenicity of the newly formed β cells from α cells is also an important aspect that needs to be addressed before applying this therapeutic strategy in type 1 diabetes. Xiao et al. have shown that *pdx1* and *MafA* gene therapy maintained euglycemia in NOD mice for four months, suggesting that the newly formed β cells are not quickly recognized by the autoimmune response [74]. Thus, this gene therapy could be combined with immunomodulation or immunosuppression to prolong the lifespan of the newly formed β-like cells.

Studies that examine the efficacy of this therapeutic strategy in treating type 2 diabetes in animal models are still lacking. In both impaired glucose tolerance (prediabetes) and T2D, insulin resistance at the hepatic level and peripheral tissues occurs due to impaired insulin signaling [2,77] and inappropriate hyperglucagonemia [78,79]. With long-standing insulin resistance, there is an eventual decline in β cell mass and function [8]. Considering that the pathophysiology of type 2 diabetes involves a decrease in insulin secretion secondary to decreased β cell mass and function, and inappropriate hyperglucagonemia, the reprogramming of α cells into insulin-secreting cells seems to be an appealing treatment for this disorder by restoring β cell mass, leading to increased insulin secretion, and by decreasing α cell mass with a potential reduction in the hyperglucagonemia and insulin resistance. Ideally, such a treatment for type 2 diabetes, with the reprogramming of α cells into insulin-producing cells, would be accompanied by lifestyle modification. Otherwise, the persistent chronic exposure of the newly formed β cells to insulin resistance, glucotoxicity and lipotoxicity would likely cause failure of the new β-like cells, with recurrence of hyperglycemia [80]. Thus, more studies are needed to address the potential benefit of using α cell to β cell conversion in treating type 2 diabetes.

Overall, further studies are required to develop and optimize this promising therapeutic strategy given the despirate need to find novel treatments and perhaps a cure for diabetes, a major health and economic problem.

## Perspectives

- Importance in the field: Diabetes is a major health and economic problem in the United States and around the world. There is currently no cure for diabetes. Converting α cells into insulin-producing cells could provide a promising therapeutic strategy to cure diabetes.
- Current thinking: The use of viral gene therapy to drive the expression of *pdx1* and *MafA* in α cells, transforming them into functional β cells, has recently become the main direction in this research field. This technique will replace the lost β cell mass and restore insulin secretion capacity in individuals with diabetes.
- Future directions: In this field, future directions include: (1) Developing a proper viral vector that could carry the genes and targets human α cells with high affinity *in vivo.* (2) Immunomodulation to prevent the immune-mediated destruction of the newly formed β cells in type 1 diabetes. (3) Investigate the potential efficacy of this approach as a therapeutic stagey for type 2 diabetes.

## Abbreviations

AAV: adeno-associated virus
NOD: non-obese diabetic
PMN: *pdx1, MafA*, and *ngn3*
SCID: severe combined immunodeficient

## References

[1] Atkinson, M.A., Eisenbarth, G.S. and Michels, A.W. (2014) Type 1 diabetes. Lancet 383, 69–82 10.1016/S0140-6736(13)60591-7 23890997 PMC4380133

[2] Defronzo, R.A. (2009) Banting Lecture. From the triumvirate to the ominous octet: a new paradigm for the treatment of type 2 diabetes mellitus. Diabetes 58, 773–795 10.2337/db09-9028 19336687 PMC2661582

[3] Rahier, J., Guiot, Y., Goebbels, R.M., Sempoux, C. and Henquin, J.C. (2008) Pancreatic beta-cell mass in European subjects with type 2 diabetes. Diabetes Obes. Metab. 10, 32–42 10.1111/j.1463-1326.2008.00969.x 18834431

[4] Yoon, K.H., Ko, S.H., Cho, J.H., Lee, J.M., Ahn, Y.B., Song, K.H. et al. (2003) Selective beta-cell loss and alpha-cell expansion in patients with type 2 diabetes mellitus in Korea. J. Clin. Endocrinol. Metab. 88, 2300–2308 10.1210/jc.2002-020735 12727989

[5] Butler, A.E., Janson, J., Bonner-Weir, S., Ritzel, R., Rizza, R.A. and Butler, P.C. (2003) Beta-cell deficit and increased beta-cell apoptosis in humans with type 2 diabetes. Diabetes 52, 102–110 10.2337/diabetes.52.1.102 12502499

[6] Weir, G.C., Butler, P.C. and Bonner-Weir, S. (2021) The beta-cell glucose toxicity hypothesis: attractive but difficult to prove. Metabolism 124, 154870 10.1016/j.metabol.2021.154870 34480921 PMC8530963

[7] Rege, N.K., Liu, M., Yang, Y., Dhayalan, B., Wickramasinghe, N.P., Chen, Y.S. et al. (2020) Evolution of insulin at the edge of foldability and its medical implications. Proc. Natl Acad. Sci. U.S.A. 117, 29618–29628 10.1073/pnas.2010908117 33154160 PMC7703552

[8] Gastaldelli, A., Ferrannini, E., Miyazaki, Y., Matsuda, M. and DeFronzo, R.A. (2004) San Antonio metabolism s. Beta-cell dysfunction and glucose intolerance: results from the San Antonio metabolism (SAM) study. Diabetologia 47, 31–39 10.1007/s00125-003-1263-9 14666364

[9] Abdul-Ghani, M.A., Jenkinson, C.P., Richardson, D.K., Tripathy, D. and DeFronzo, R.A. (2006) Insulin secretion and action in subjects with impaired fasting glucose and impaired glucose tolerance: results from the Veterans Administration Genetic Epidemiology Study. Diabetes 55, 1430–1435 10.2337/db05-1200 16644701

[10] Weyer, C., Bogardus, C., Mott, D.M. and Pratley, R.E. (1999) The natural history of insulin secretory dysfunction and insulin resistance in the pathogenesis of type 2 diabetes mellitus. J. Clin. Invest. 104, 787–794 10.1172/JCI7231 10491414 PMC408438

[11] Del Prato, S. (2003) Loss of early insulin secretion leads to postprandial hyperglycaemia. Diabetologia 46, M2–M8 10.1007/s00125-002-0930-6 12652352

[12] Aguilar, R.B. (2011) Evaluating treatment algorithms for the management of patients with type 2 diabetes mellitus: a perspective on the definition of treatment success. Clin. Ther. 33, 408–424 10.1016/j.clinthera.2011.04.008 21635988

[13] Godinho, R., Mega, C., Teixeira-de-Lemos, E., Carvalho, E., Teixeira, F., Fernandes, R. et al. (2015) The place of dipeptidyl peptidase-4 inhibitors in type 2 diabetes therapeutics: a “me too” or “the special one” antidiabetic class? J. Diabetes Res. 2015, 806979 10.1155/2015/806979 26075286 PMC4449938

[14] Marin-Penalver, J.J., Martin-Timon, I., Sevillano-Collantes, C. and Del Canizo-Gomez, F.J. (2016) Update on the treatment of type 2 diabetes mellitus. World J. Diabetes 7, 354–395 10.4239/wjd.v7.i17.354 27660695 PMC5027002

[15] Herrera, P.L. (2000) Adult insulin- and glucagon-producing cells differentiate from two independent cell lineages. Development 127, 2317–2322 10.1242/dev.127.11.2317 10804174

[16] Teitelman, G., Alpert, S., Polak, J.M., Martinez, A. and Hanahan, D. (1993) Precursor cells of mouse endocrine pancreas coexpress insulin, glucagon and the neuronal proteins tyrosine hydroxylase and neuropeptide Y, but not pancreatic polypeptide. Development 118, 1031–1039 10.1242/dev.118.4.1031 7903631

[17] Herrera, P.L., Huarte, J., Sanvito, F., Meda, P., Orci, L. and Vassalli, J.D. (1991) Embryogenesis of the murine endocrine pancreas; early expression of pancreatic polypeptide gene. Development 113, 1257–1265 10.1242/dev.113.4.1257 1811941

[18] Gu, G., Dubauskaite, J. and Melton, D.A. (2002) Direct evidence for the pancreatic lineage: NGN3+ cells are islet progenitors and are distinct from duct progenitors. Development 129, 2447–2457 10.1242/dev.129.10.2447 11973276

[19] Guz, Y., Montminy, M.R., Stein, R., Leonard, J., Gamer, L.W., Wright, C.V. et al. (1995) Expression of murine STF-1, a putative insulin gene transcription factor, in beta cells of pancreas, duodenal epithelium and pancreatic exocrine and endocrine progenitors during ontogeny. Development 121, 11–18 10.1242/dev.121.1.11 7867492

[20] Ohlsson, H., Karlsson, K. and Edlund, T. (1993) IPF1, a homeodomain-containing transactivator of the insulin gene. EMBO J. 12, 4251–4259 10.1002/j.1460-2075.1993.tb06109.x 7901001 PMC413720

[21] Hani, E.H., Stoffers, D.A., Chevre, J.C., Durand, E., Stanojevic, V., Dina, C. et al. (1999) Defective mutations in the insulin promoter factor-1 (IPF-1) gene in late-onset type 2 diabetes mellitus. J. Clin. Invest. 104, R41–R48 10.1172/JCI7469 10545531 PMC409821

[22] Stoffers, D.A., Ferrer, J., Clarke, W.L. and Habener, J.F. (1997) Early-onset type-II diabetes mellitus (MODY4) linked to IPF1. Nat. Genet. 17, 138–139 10.1038/ng1097-138 9326926

[23] Gannon, M., Ables, E.T., Crawford, L., Lowe, D., Offield, M.F., Magnuson, M.A. et al. (2008) . pdx-1 function is specifically required in embryonic beta cells to generate appropriate numbers of endocrine cell types and maintain glucose homeostasis. Dev. Biol. 314, 406–417 10.1016/j.ydbio.2007.10.038 18155690 PMC2269701

[24] Ahlgren, U., Jonsson, J., Jonsson, L. and Simu, K. (1998) Edlund H. beta-cell-specific inactivation of the mouse Ipf1/Pdx1 gene results in loss of the beta-cell phenotype and maturity onset diabetes. Genes Dev. 12, 1763–1768 10.1101/gad.12.12.1763 9637677 PMC316911

[25] Gradwohl, G., Dierich, A., LeMeur, M. and Guillemot, F. (2000) neurogenin3 is required for the development of the four endocrine cell lineages of the pancreas. Proc. Natl Acad. Sci. U.S.A. 97, 1607–1611 10.1073/pnas.97.4.1607 10677506 PMC26482

[26] Schwitzgebel, V.M., Scheel, D.W., Conners, J.R., Kalamaras, J., Lee, J.E., Anderson, D.J. et al. (2000) Expression of neurogenin3 reveals an islet cell precursor population in the pancreas. Development 127, 3533–3542 10.1242/dev.127.16.3533 10903178

[27] Jenny, M., Uhl, C., Roche, C., Duluc, I., Guillermin, V., Guillemot, F. et al. (2002) Neurogenin3 is differentially required for endocrine cell fate specification in the intestinal and gastric epithelium. EMBO J. 21, 6338–6347 10.1093/emboj/cdf649 12456641 PMC136953

[28] Sheets, T.P., Park, K.E., Park, C.H., Swift, S.M., Powell, A., Donovan, D.M. et al. (2018) Targeted mutation of NGN3 gene disrupts pancreatic endocrine cell development in pigs. Sci. Rep. 8, 3582 10.1038/s41598-018-22050-0 29483633 PMC5827570

[29] Rukstalis, J.M. and Habener, J.F. (2009) Neurogenin3: a master regulator of pancreatic islet differentiation and regeneration. Islets 1, 177–184 10.4161/isl.1.3.9877 21099270

[30] Vanhoose, A.M., Samaras, S., Artner, I., Henderson, E., Hang, Y. and Stein, R. (2008) MafA and MafB regulate Pdx1 transcription through the Area II control region in pancreatic beta cells. J. Biol. Chem. 283, 22612–22619 10.1074/jbc.M802902200 18522939 PMC2504898

[31] Artner, I., Hang, Y., Mazur, M., Yamamoto, T., Guo, M., Lindner, J. et al. (2010) MafA and MafB regulate genes critical to beta-cells in a unique temporal manner. Diabetes 59, 2530–2539 10.2337/db10-0190 20627934 PMC3279542

[32] Hang, Y. and Stein, R. (2011) MafA and MafB activity in pancreatic beta cells. Trends Endocrinol. Metab. 22, 364–373 10.1016/j.tem.2011.05.003 21719305 PMC3189696

[33] Nishimura, W., Bonner-Weir, S. and Sharma, A. (2009) Expression of MafA in pancreatic progenitors is detrimental for pancreatic development. Dev. Biol. 333, 108–120 10.1016/j.ydbio.2009.06.029 19576197 PMC2737322

[34] Conrad, E., Dai, C., Spaeth, J., Guo, M., Cyphert, H.A., Scoville, D. et al. (2016) The MAFB transcription factor impacts islet alpha-cell function in rodents and represents a unique signature of primate islet beta-cells. Am. J. Physiol. Endocrinol. Metab. 310, E91–E102 10.1152/ajpendo.00285.2015 26554594 PMC4675799

[35] Artner, I., Blanchi, B., Raum, J.C., Guo, M., Kaneko, T., Cordes, S. et al. (2007) MafB is required for islet beta cell maturation. Proc. Natl Acad. Sci. U.S.A. 104, 3853–3858 10.1073/pnas.0700013104 17360442 PMC1803762

[36] Rall, L.B., Pictet, R.L., Williams, R.H. and Rutter, W.J. (1973) Early differentiation of glucagon-producing cells in embryonic pancreas: a possible developmental role for glucagon. Proc. Natl Acad. Sci. U.S.A. 70, 3478–3482 10.1073/pnas.70.12.3478 4519640 PMC427263

[37] Murtaugh, L.C. (2007) Pancreas and beta-cell development: from the actual to the possible. Development 134, 427–438 10.1242/dev.02770 17185316

[38] Cozar-Castellano, I. and Stewart, A.F. (2005) Molecular engineering human hepatocytes into pancreatic beta cells for diabetes therapy. Proc. Natl Acad. Sci. U.S.A. 102, 7781–7782 10.1073/pnas.0503261102 15911749 PMC1142398

[39] Hao, E., Tyrberg, B., Itkin-Ansari, P., Lakey, J.R., Geron, I., Monosov, E.Z. et al. (2006) Beta-cell differentiation from nonendocrine epithelial cells of the adult human pancreas. Nat. Med. 12, 310–316 10.1038/nm1367 16491084

[40] McKimpson, W.M. and Accili, D. (2019) Reprogramming cells to make insulin. J. Endocr. Soc. 3, 1214–1226 10.1210/js.2019-00040 31187080 PMC6546342

[41] Li, W., Nakanishi, M., Zumsteg, A., Shear, M., Wright, C., Melton, D.A. et al. (2014) *In vivo* reprogramming of pancreatic acinar cells to three islet endocrine subtypes. eLife 3, e01846 10.7554/eLife.01846 24714494 PMC3977343

[42] Zhou, Q., Brown, J., Kanarek, A., Rajagopal, J. and Melton, D.A. (2008) *In vivo* reprogramming of adult pancreatic exocrine cells to beta-cells. Nature 455, 627–632 10.1038/nature07314 18754011 PMC9011918

[43] Sapir, T., Shternhall, K., Meivar-Levy, I., Blumenfeld, T., Cohen, H., Skutelsky, E. et al. (2005) Cell-replacement therapy for diabetes: generating functional insulin-producing tissue from adult human liver cells. Proc. Natl Acad. Sci. U.S.A. 102, 7964–7969 10.1073/pnas.0405277102 15899968 PMC1142350

[44] Ferber, S., Halkin, A., Cohen, H., Ber, I., Einav, Y., Goldberg, I. et al. (2000) Pancreatic and duodenal homeobox gene 1 induces expression of insulin genes in liver and ameliorates streptozotocin-induced hyperglycemia. Nat. Med. 6, 568–72 10.1038/75050 10802714

[45] Aviv, V., Meivar-Levy, I., Rachmut, I.H., Rubinek, T., Mor, E. and Ferber, S. (2009) Exendin-4 promotes liver cell proliferation and enhances the PDX-1-induced liver to pancreas transdifferentiation process. J. Biol. Chem. 284, 33509–33520 10.1074/jbc.M109.017608 19755420 PMC2785195

[46] Cim, A., Sawyer, G.J., Zhang, X., Su, H., Collins, L., Jones, P. et al. (2012) *In vivo* studies on non-viral transdifferentiation of liver cells towards pancreatic beta cells. J. Endocrinol. 214, 277–288 10.1530/JOE-12-0033 22685335

[47] Banga, A., Akinci, E., Greder, L.V., Dutton, J.R. and Slack, J.M. (2012) *In vivo* reprogramming of Sox9+ cells in the liver to insulin-secreting ducts. Proc. Natl Acad. Sci. U.S.A. 109, 15336–15341 10.1073/pnas.1201701109 22949652 PMC3458366

[48] Banga, A., Greder, L.V., Dutton, J.R. and Slack, J.M. (2014) Stable insulin-secreting ducts formed by reprogramming of cells in the liver using a three-gene cocktail and a PPAR agonist. Gene Ther. 21, 19–27 10.1038/gt.2013.50 24089243 PMC3880604

[49] Barker, N., van Es, J.H., Kuipers, J., Kujala, P., van den Born, M., Cozijnsen, M. et al. (2007) Identification of stem cells in small intestine and colon by marker gene Lgr5. Nature 449, 1003–1007 10.1038/nature06196 17934449

[50] Barker, N., Huch, M., Kujala, P., van de Wetering, M., Snippert, H.J., van Es, J.H. et al. (2010) Lgr5(+ve) stem cells drive self-renewal in the stomach and build long-lived gastric units in vitro. Cell Stem Cell 6, 25–36 10.1016/j.stem.2009.11.013 20085740

[51] Talchai, C., Xuan, S., Kitamura, T., DePinho, R.A. and Accili, D. (2012) Generation of functional insulin-producing cells in the gut by Foxo1 ablation. Nat. Genet. 44, 406–412. S1 10.1038/ng.2215 22406641 PMC3315609

[52] Bouchi, R., Foo, K.S., Hua, H., Tsuchiya, K., Ohmura, Y., Sandoval, P.R. et al. (2014) FOXO1 inhibition yields functional insulin-producing cells in human gut organoid cultures. Nat. Commun. 5, 4242 10.1038/ncomms5242 24979718 PMC4083475

[53] Chen, Y.J., Finkbeiner, S.R., Weinblatt, D., Emmett, M.J., Tameire, F., Yousefi, M. et al. (2014) De novo formation of insulin-producing “neo-beta cell islets” from intestinal crypts. Cell Rep. 6, 1046–1058 10.1016/j.celrep.2014.02.013 24613355 PMC4245054

[54] Ariyachet, C., Tovaglieri, A., Xiang, G., Lu, J., Shah, M.S., Richmond, C.A. et al. (2016) Reprogrammed stomach tissue as a renewable source of functional beta cells for blood glucose regulation. Cell Stem Cell 18, 410–421 10.1016/j.stem.2016.01.003 26908146 PMC4779391

[55] Gomez, D.L., O'Driscoll, M., Sheets, T.P., Hruban, R.H., Oberholzer, J., McGarrigle, J.J. et al. (2015) Neurogenin 3 expressing cells in the human exocrine pancreas have the capacity for endocrine cell fate. PLoS ONE 10, e0133862 10.1371/journal.pone.0133862 26288179 PMC4545947

[56] Bonal, C. and Herrera, P.L. (2008) Genes controlling pancreas ontogeny. Int. J. Dev. Biol. 52, 823–835 10.1387/ijdb.072444cb 18956314

[57] Thorel, F., Nepote, V., Avril, I., Kohno, K., Desgraz, R., Chera, S. et al. (2010) Conversion of adult pancreatic alpha-cells to beta-cells after extreme beta-cell loss. Nature 464, 1149–1154 10.1038/nature08894 20364121 PMC2877635

[58] Wei, R., Gu, L., Yang, J., Yang, K., Liu, J., Le, Y. et al. (2019) Antagonistic glucagon receptor antibody promotes alpha-cell proliferation and increases beta-cell mass in diabetic mice. iScience 16, 326–339 10.1016/j.isci.2019.05.030 31203188 PMC6581654

[59] Quesada, I., Tuduri, E., Ripoll, C. and Nadal, A. (2008) Physiology of the pancreatic alpha-cell and glucagon secretion: role in glucose homeostasis and diabetes. J. Endocrinol. 199, 5–19 10.1677/JOE-08-0290 18669612

[60] Zhang, Q., Ramracheya, R., Lahmann, C., Tarasov, A., Bengtsson, M., Braha, O. et al. (2013) Role of KATP channels in glucose-regulated glucagon secretion and impaired counterregulation in type 2 diabetes. Cell Metab. 18, 871–882 10.1016/j.cmet.2013.10.014 24315372 PMC3851686

[61] Salvioli, B., Bovara, M., Barbara, G., De Ponti, F., Stanghellini, V., Tonini, M. et al. (2002) Neurology and neuropathology of the pancreatic innervation. JOP 3, 26–33 PMID:11884764

[62] Pipeleers, D., Keymeulen, B., Chatenoud, L., Hendrieckx, C., Ling, Z., Mathieu, C. et al. (2002) A view on beta cell transplantation in diabetes. Ann. N. Y. Acad. Sci. 958, 69–76 10.1111/j.1749-6632.2002.tb02948.x 12021085

[63] Steiner, D.J., Kim, A., Miller, K. and Hara, M. (2010) Pancreatic islet plasticity: interspecies comparison of islet architecture and composition. Islets 2, 135–145 10.4161/isl.2.3.11815 20657742 PMC2908252

[64] Shiota, C., Prasadan, K., Guo, P., El-Gohary, Y., Wiersch, J., Xiao, X. et al. (2013) . alpha-Cells are dispensable in postnatal morphogenesis and maturation of mouse pancreatic islets. Am. J. Physiol. Endocrinol. Metab. 305, E1030–E1040 10.1152/ajpendo.00022.2013 23982158

[65] Collombat, P., Xu, X., Ravassard, P., Sosa-Pineda, B., Dussaud, S., Billestrup, N. et al. (2009) The ectopic expression of Pax4 in the mouse pancreas converts progenitor cells into alpha and subsequently beta cells. Cell 138, 449–462 10.1016/j.cell.2009.05.035 19665969 PMC2792203

[66] Sangan, C.B., Jover, R., Heimberg, H. and Tosh, D. (2015) *In vitro* reprogramming of pancreatic alpha cells towards a beta cell phenotype following ectopic HNF4alpha expression. Mol. Cell. Endocrinol. 399, 50–59 10.1016/j.mce.2014.09.009 25224487

[67] Zhang, Y., Fava, G.E., Wang, H., Mauvais-Jarvis, F., Fonseca, V.A. and Wu, H. (2016) PAX4 gene transfer induces alpha-to-beta cell phenotypic conversion and confers therapeutic benefits for diabetes treatment. Mol. Ther. 24, 251–260 10.1038/mt.2015.181 26435408 PMC4817809

[68] Chakravarthy, H., Gu, X., Enge, M., Dai, X., Wang, Y., Damond, N. et al. (2017) Converting adult pancreatic islet alpha cells into beta cells by targeting both Dnmt1 and Arx. Cell Metab. 25, 622–634 10.1016/j.cmet.2017.01.009 28215845 PMC5358097

[69] Li, J., Casteels, T., Frogne, T., Ingvorsen, C., Honore, C., Courtney, M. et al. (2017) Artemisinins target GABAA receptor signaling and impair alpha cell identity. Cell 168, 86–100 e15 10.1016/j.cell.2016.11.010 27916275 PMC5236063

[70] van der Meulen, T., Lee, S., Noordeloos, E., Donaldson, C.J., Adams, M.W., Noguchi, G.M. et al. (2018) Artemether does not turn alpha cells into beta cells. Cell Metab. 27, 218–225 e4 10.1016/j.cmet.2017.10.002 29103923 PMC5762275

[71] Ben-Othman, N., Vieira, A., Courtney, M., Record, F., Gjernes, E., Avolio, F. et al. (2017) Long-term GABA administration induces alpha cell-mediated beta-like cell neogenesis. Cell 168, 73–85 e11 10.1016/j.cell.2016.11.002 27916274

[72] Lee, Y.S., Shin, S., Shigihara, T., Hahm, E., Liu, M.J., Han, J. et al. (2007) Glucagon-like peptide-1 gene therapy in obese diabetic mice results in long-term cure of diabetes by improving insulin sensitivity and reducing hepatic gluconeogenesis. Diabetes 56, 1671–1679 10.2337/db06-1182 17369525

[73] Matsuoka, T.A., Kawashima, S., Miyatsuka, T., Sasaki, S., Shimo, N., Katakami, N. et al. (2017) Mafa enables Pdx1 to effectively convert pancreatic islet progenitors and committed islet alpha-cells into beta-cells *in vivo*. Diabetes 66, 1293–1300 10.2337/db16-0887 28223284 PMC5399608

[74] Xiao, X., Guo, P., Shiota, C., Zhang, T., Coudriet, G.M., Fischbach, S. et al. (2018) Endogenous reprogramming of alpha cells into beta cells, induced by viral gene therapy, reverses autoimmune diabetes. Cell Stem Cell 22, 78–90 e4 10.1016/j.stem.2017.11.020 29304344 PMC5757249

[75] Furuyama, K., Chera, S., van Gurp, L., Oropeza, D., Ghila, L., Damond, N. et al. (2019) Diabetes relief in mice by glucose-sensing insulin-secreting human alpha-cells. Nature 567, 43–48 10.1038/s41586-019-0942-8 30760930 PMC6624841

[76] Korpos, E., Kadri, N., Kappelhoff, R., Wegner, J., Overall, C.M., Weber, E. et al. (2013) The peri-islet basement membrane, a barrier to infiltrating leukocytes in type 1 diabetes in mouse and human. Diabetes 62, 531–542 10.2337/db12-0432 23139348 PMC3554379

[77] Henry, R.R., Wallace, P. and Olefsky, J.M. (1986) Effects of weight loss on mechanisms of hyperglycemia in obese non-insulin-dependent diabetes mellitus. Diabetes 35, 990–998 10.2337/diab.35.9.990 3527829

[78] DeFronzo, R.A. and Ferrannini, E. (1987) Regulation of hepatic glucose metabolism in humans. Diabetes Metab. Rev. 3, 415–459 10.1002/dmr.5610030204 3552529

[79] Consoli, A., Nurjhan, N., Reilly, Jr, J.J., Bier, D.M. and Gerich, J.E. (1990) Mechanism of increased gluconeogenesis in noninsulin-dependent diabetes mellitus. Role of alterations in systemic, hepatic, and muscle lactate and alanine metabolism. J. Clin. Invest. 86, 2038–2045 10.1172/JCI114940 2254458 PMC329842

[80] Kahn, S.E., Hull, R.L. and Utzschneider, K.M. (2006) Mechanisms linking obesity to insulin resistance and type 2 diabetes. Nature 444, 840–846 10.1038/nature05482 17167471

