# Supplementary material for: Alpha-to-beta cell trans-differentiation for treatment of diabetes
Source: Biochem Soc Trans. 2021 Dec 9;49(6):2539–48. doi: 10.1042/BST20210244 (PMC8786296; doi:10.1042/BST20210244)
Supplement: Supplementary Information [file BST-49-2539-s1.pdf]

Funding: This work was partially supported by NIDDK funding to GG (RO1DK111460).
